# Acute Ischemic Stroke and COVID-19: Experience From a Comprehensive Stroke Center in Midwest US

**DOI:** 10.3389/fneur.2020.00910

**Published:** 2020-08-20

**Authors:** Parneet Grewal, Pranusha Pinna, Julianne P. Hall, Rima M. Dafer, Tachira Tavarez, Danielle R. Pellack, Rajeev Garg, Nicholas D. Osteraas, Alejandro Vargas, Sayona John, Ivan Da Silva, James J. Conners

**Affiliations:** Department of Neurological Sciences, Rush University Medical Center, Chicago, IL, United States

**Keywords:** acute ischemic stroke, COVID-19, racial disparity, coronavirus, stroke care

## Abstract

**Background:** COVID-19 has been associated with increased risk of venous and arterial thromboembolism including ischemic stroke. We report on patients with acute ischemic stroke and concomitant COVID-19 in a diverse patient population.

**Methods:** This is a retrospective analysis of patients hospitalized with acute ischemic stroke (AIS) and COVID-19 to our comprehensive stroke center in Chicago, IL, between March 1, 2020, and April 30, 2020. We reviewed stroke characteristics, etiologies, and composite outcomes. We then compared our cohort with historic patients with AIS without COVID-19 admitted in the same time frame in 2019 and 2020.

**Results:** Out of 13 patients with AIS and COVID-19, Latinos and African-Americans compromised the majority of our cohort (76.8%), with age ranging from 31–80 years. Most strokes were cortical (84.6%) and more than 50% of patients had no identifiable source, and were categorized as embolic stroke of unknown source (ESUS). A trend toward less alteplase administration was noted in the COVID-19 stroke patients compared to the non-COVID group from 2020 and 2019 (7.1 vs. 20.7% *p* 0.435 and 7.1 vs. 27.2% *p* 0.178). Endovascular thrombectomy was performed in 3 (23%) patients. Systemic thrombotic complications occurred in 3 (23%) COVID-19 AIS patients. Median National Institutes of Health Stroke Scale and modified Rankin Scale at discharge were 11 (IQR 4–23) and 4 (IQR 3–4), respectively. In the logistic regression model corrected for age and sex, COVID-19 was associated with discharge to mRS > 2 (*p* 0.046, OR 3.82, CI 1.02–14.3). Eight patients (63.8%) were discharged home or to acute rehabilitation, and two deceased from COVID-19 complications.

**Conclusion:** AIS in the setting of COVID-19 is associated with worse outcomes, especially among African-American and Latino populations. Large vessel disease with ESUS was common suggesting an increased risk of coagulopathy and endothelial dysfunction as a potential etiology.

## Introduction

The severe acute respiratory distress syndrome virus (SARS-CoV-2) causing the coronavirus disease 2019 (COVID-19) was first identified in Wuhan, China, and has since spread throughout the world at an alarming rate, affecting over 7 million people as of June 7, 2020 (1). Neurologic involvement including stroke has been reported (2, 3). Ischemic strokes in COVID-19 have been associated with poor outcomes but the data are mainly limited to Asian and white populations. Data on the potential increased risk of stroke in COVID-19 has not yet been reported in racially diverse patient populations such as Latinos and African-Americans (4, 5).

In this manuscript, we report clinical and laboratory characteristics along with outcomes of patients with COVID-19 and acute ischemic stroke (AIS) who presented to our comprehensive stroke center in Chicago, IL, between March 1, 2020 and April 30, 2020. Our tertiary care center has been in the epicenter of the outbreak in Chicago in the Midwest US and regularly cares for an underserved and diverse patient population with lower health literacy. To validate our findings, to further identify mechanisms of stroke and outcome variables, we compared our cohort with stroke patients from the same time frame in 2020 along with historical cohort from 2019.

## Methods

We conducted a retrospective observational analysis of the medical records of all patients admitted to Rush University Medical Center in Chicago, Illinois, United States, between March 1, 2020 and April 30, 2020, with the diagnosis of AIS, confirmed on magnetic resonance imaging (MRI) or computed tomography (CT) and who were positive for COVID-19 with real-time reverse transcriptase polymerase-chain-reaction assay from a nasopharyngeal swab. To compensate for any seasonal or monthly variation in incidence and mortality from AIS, we compared the cohort with a control group of non-COVID-19 AIS patients hospitalized within the matched time frame in 2020. We also compared with a historical cohort from 2019 to control for any changes in the patient population over time. Two different cohorts were used as control to avoid random variation in demographics between pre-COVID and COVID-era.

Demographics and clinical and laboratory data were collected via a review of the electronic medical record system. These included age, gender, ethnicity, pre-existing vascular risk factors, admission vital signs, laboratory values, and National Institutes of Health Stroke Scale (NIHSS) score on admission and at discharge (or at the time of data collection for patients still hospitalized). We divided the patients with COVID-19 AIS into the “COVID” group, defined as patients admitted initially with COVID-19 symptoms then subsequently developing AIS, and the “neuro” group, with patients admitted for AIS as initial symptoms, and tested positive for COVID-19. “COVID” group had more extensive inflammatory and coagulopathy workup. All patients received acute stroke care per the American Heart Association and American Stroke Association guidelines (6).

We used the (TOAST) classification to determine stroke etiology (7). All AIS patients received extensive evaluation including advanced cardiac imaging, hypercoagulability panel, and prolonged cardiac monitoring while admitted inpatient. We further evaluated cryptogenic stroke patients to identify embolic stroke of unknown source (ESUS) etiology according to the published criteria (8). Patients with potential stroke mechanisms thought to be due to hypercoagulable state due to COVID-19 were placed under cryptogenic and/or ESUS mechanisms.

Outcome measures were based on discharge disposition and modified Rankin Scale (mRS) (9). COVID-19 severity was defined as mild, regular, or severe/critical based on the 7th edition of “Novel Coronavirus Pneumonia Diagnosis and Treatment Plan,” with the description as follows: mild, defined as minor clinical symptoms and lack of lung inflammation on imaging; regular, with fever and respiratory tract symptoms, and evidence of visible lung inflammation on imaging; severe, with either shortness of breath, RR more than 30 breaths per minute, or SpO2 <93% at rest on pulse oximetry; and critical, with the need for mechanical ventilation or the presence of shock or combined failure of other organs requiring ICU monitoring (10).

Statistical testing was used to detect in-between group differences and association of individual variables to the pre-selected outcomes. The cohort groups were compared using Student's *t*-test for parametric continuous variables, MannWhitney U test for non-parametric continuous variables, and Fisher's exact test for dichotomous variables. Logistic regression was used to analyze selected variables (either clinically relevant or with statistical association in the first analysis) in regards to the pre-selected outcome measurements, correcting for confounding factors. All analyses were performed using commercially available SPSS (v. 21, Chicago IL, USA) statistical software. Significance was set at *p* < 0.05. Data were collected using REDCap, an electronic data capture tool hosted at our institution (11). This research protocol was approved by the Rush University institutional review board.

## Results

### Demographics and Clinical Characteristics

Between March 1, 2020 and April 30, 2020, ~650 patients were hospitalized with COVID-19, of whom 13 patients had AIS (estimated percentage of 2.0%). The COVID-19 AIS cohort was mostly comprised of Latino (46.1%) and African-American (30.7%) individuals, ages ranging from 31 to 80 years (mean 61.6 years). There were 6 patients in the “COVID” group (47%) and 7 in the “Neuro” group (53%). The average time for diagnosis of AIS in the “COVID” group after the hospitalization was 7.1 ± 5.1 days. Conventional vascular risk factors were common in both with no specific predilection for either the “COVID” or the “Neuro” groups. The three most common risk factors in the COVID-19 AIS cohort were hypertension (69.2%), type 2 diabetes mellitus (DM) (69.2%), and hyperlipidemia (30.7%). The COVID-19 was considered severe or critical in 61.5% (*n* = 8) divided between the “COVID” (50%) and the “Neuro” (71.4%) groups, and mild and/or regular in 38.5% (*n* = 5) of patients. Out of the 13 patients, 30.7% (*n* = 4) patients also had superimposed bacterial infection. Median admission NIHSS was 16 (IQR 4–23) in all the COVID-AIS patients, with higher score of 13 in the “Neuro” group compared to 4.5 in the “COVID” group (Table 1).

**Table 1:** Demographics and clinical features of 13 consecutive acute ischemic stroke patients with COVID-19 infection.

**Patient**	**1**	**2**	**3**	**4**	**5**	**6**	**7**	**8**	**9**	**10**	**11**	**12**	**13**
Presenting symptoms (COVID vs. Neuro)	COVID	COVID	COVID	COVID	COVID	COVID	Neuro	Neuro	Neuro	Neuro	Neuro	Neuro	Neuro
Time of onset of neuro symptoms	(2 days)	(15 days)	(3 days)	(11 days)	(4 days)	(8 days)							
Age range	55–60	70–75	55–60	75–80	60–65	70–74	35–40	65–70	80–85	60–65	45–50	80–85	30–35
Race/Ethnicity	AA	Other	Latino	Other	AA	AA	White	Latino	Latino	AA	Latino	Latino	Latino
Vascular risk factors	None	HTN, DM2, CAD	HTN, HLD, DM2	HTN, Afib	HTN, Obesity	HTN, DM2	DM2	HTN, DM2 Obesity	DM2	HTN, HLD, DM2, CAD	HTN, HLD, DM2	HTN, HLD	None
Severity of COVID-19^*^	Regular	Severe/critical	Severe/critical	Severe/critical	Mild	Mild	Mild	Severe/critical	Severe/critical	Severe/critical	Severe/critical	Regular	Severe/critical
Admission GCS	15	15	13	12	14	11	15	13	14	6	5	15	14
NIHSS on admission	2	2	23	6	3	23	4	26	4	28	22	13	11
NIHSS on discharge or last NIHSS on exam	2	4	13	14	2	18	6	3	28	19	35	0	16
mRS on discharge or last mRS on exam	2	3	4	5	2	3	4	3	6	4	6	0	4
Stroke etiology	Cryptogenic	SW disease	Cryptogenic	Cardio-embolism (Afib)	Cardio-embolism (MI <4 weeks)	Large artery atherosclerosis (ICA)	Cryptogenic	Cryptogenic	Cryptogenic	Cardio-embolism (PFO with *in situ* DVT)	Large artery atherosclerosis (ICA)	Cryptogenic	Cryptogenic
	ESUS		ESUS				ESUS	ESUS	ESUS			ESUS	ESUS
Stroke location	Cortical	Subcortical	Cortical	Cortical	Cortical	Cortical	Cortical	Cortical	Cortical	Cortical	Cortical	Cortical	Brainstem and cerebellum
	Bilateral, multifocal	Left MCA	Left MCA	Right MCA	Right MCA	Right MCA	Left MCA	Left PCA	Left PCA, right MCA	Left PCA left MCA	Left MCA	Right MCA	
Large vessel occlusion	No	No	Yes	No	Yes	Yes	Yes	No	No	No	Yes	No	No
Acute intervention	No	No	Thrombectomy/TICI2B	No	No	No	Thrombectomy/TICI2B	No	No	No	IV-tPAthrombectomy/TICI3	IV-tPA	No
Systemic arterial or venous thrombosis	No	No	Arterial	No	No	No	No	No	No	Deep venous	No	No	Arterial
Disseminated intravascular coagulopathy	No	No	No	Yes	No	No	No	No	No	No	No	No	No
Therapeutic anticoagulation	None	None	Enoxaparin	Apixaban	None	None	Apixaban	None	None	Rivaroxaban	None	Coumadin	Enoxaparin
Antiplatelets	None	Aspirin	None	Aspirin	Ticagrelor aspirin	Aspirin	None	Aspirin	Aspirin	None	None	Clopidogrel	None
Discharge disposition	Home	Acute rehab	Acute rehab	LTAC	Home	Acute rehab	Acute rehab	Acute rehab	Expired	LTAC	Expired	Home	-

* *Severity of COVID-19 infection was based on the 7th edition of “Novel Coronavirus pneumonia diagnosis and treatment plan” and the patients were divided into mild form (clinical symptoms are minor and imaging does not show any lung inflammation), regular (has fever and respiratory tract symptoms, imaging shows visible lung inflammation), severe (adults who have either shortness of breath, RR>30 breaths/min, SpO2 <93% at rest) and critical form (mechanical ventilation required or shock or combined failure of other organs that requires ICU monitoring) (10)*.

*COVID-19, Coronavirus disease 19; F, Female; M(Male; AA, African-American; HTN, Hypertension; HLD, Hyperlipidemia; DM2, Diabetes mellitus type 2; CAD, coronary artery disease; GCS, Glasgow comma scale; NIHSS, National Institutes of Health Stroke Scale; mRS, modified Rankin scale; ESUS, Embolic stroke of unknown source; SW dis., Small vessel disease; MI, Myocardial infarction; ICA, Internal carotid artery; DVT, deep venous thrombosis; PFO, patent foramen ovale; MCA, middle cerebral artery; PCA, posterior cerebral artery; TICI, Thrombolysis in cerebral ischemia score; IV-tPA, intravenous-alteplase; LTAC, long-term acute care facility*.

### Laboratory Characteristics

Initial vitals and laboratory values are demonstrated in detail in Table 2. Average temperature was 99.6 ± 1.8 degree F, mean arterial pressure was 82.6 ± 38.7 mmHg, heart rate was 90.6 ± 29.4 beats per minute, respiratory rate was 22 ± 8 breaths per minute, and oxygen saturation was 93.3 ± 7.8%. Patients in the “COVID” group were more likely to have multiorgan failure and elevated inflammatory and coagulopathy markers (Table 2).

**Table 2:** Admission vitals and admission laboratory values for acute ischemic stroke patients with COVID-19.

**Patient**	**1**	**2**	**3**	**4**	**5**	**6**	**7**	**8**	**9**	**10**	**11**	**12**	**13**	**Mean admission values**
Presenting symptoms (COVID vs. Neuro)	COVID	COVID	COVID	COVID	COVID	COVID	Neuro	Neuro	Neuro	Neuro	Neuro	Neuro	Neuro	
Temp. (F)	102.5	100.2	99.2	95.4	97.3	97.4	98.0	99.2	98.1	101.7	97.8	98.4	98.7	99.5
MAP (mmHg)	82	75	111	96	61	107	91	113	104	111	01	6	116	82.6
HR (per minute)	128	85	118	151	49	93	68	80	87	101	76	48	94	90.6
RR (per minute)	20	36	23	10	18	20	18	24	18	38	28	13	19	21.9
SpO2 (%)	99	95	94	97	98	95	97	76	89	78	97	100	99	93.3
WBC (K/uL) (range)	0.21 (0.24–12.57)	5 (5–15.12)	19.53 (7.23–21.9)	7.82 (5.65–10.92	20.95 (11.52–24.19)	6.51 (5.80–10.19)	15.15 (6.85–15.15)	11.74 (5.86–11.91)	5.39 (5.00–11.23)	7.74 (7.74–15.05)	7.5 (7.5–22.4)	4.45 (2.49–5.21)	6.23 (6.23–15.53)	9.09
Lymphocyte count (K/uL) (range)	0.59 (0.21–0.98)	0.58 (0.53–2.62)	0.97 (0.76–1.55)	0.67 (0.54–2.17)	0.98 (0.98–1.82)	–	4.22 (4.22–3.25)	0.50 (0.58–1.58)	0.87 (0.82–0.97)	1.81 (0.59–2.6)	02.2 (0.52–2.2)	0.67 (0.44–1.16)	1.24 (1.22–1.52)	1.27
Platelet count (K/uL) (range)	4 (4–46)	187 (110–243)	468 (355–868)	83 (30–204)	277 (277–486)	309 (263–357)	348 (228–348)	219 (214–477)	293 (265–364)	284 (153–318)	173 (140–200)	224 (158–235)	292 (172–399)	243.15
AST (U/L) (range)	6 (5–152)	77 (22–113)	186 (18–256)	1023 (32–1626)	89 (19–89)	24 (24–55)	16 (12–30)	376 (24–395)	19	64 (18–78)	15 (15–162)	32 (32–129)	96 (25–96)	155.6
ALT (U/L) (range)	20 (8–262)	65 (16–96)	130 (10–144)	360 (24–649)	15 (13–17)	26 (26–68)	29 (19–31)	364 (24–364)	13	34 (25–57)	12 (8–24)	22 (22–62)	195 (105–195)	98.8
Creatinine (mg/dL) (range)	0.62 (0.49–0.67)	2.97 (1.78–4.6)	1.07 (0.77–1.12)	5.46 (1.16–5.46)	3.13 (2.16–3.29)	2.39 (2.39–2.81)	0.71 (0.51–0.71)	2.11 (0.67–2.38)	2.48 (2.05–2.48)	1.9 (0.83–3.84	3.24 (2.3–10.01)	3.05 (3.05 – 8.0)	0.66 (0.57–0.72)	2.29
D-dimer (mg/L FEU) (range)	>27.5	>27.5 (6.07–>22.50)	14.78 (2.04–14.78)	>27.50	3.79 (3.13–3.79)	3.79	0.32 (0.30–0.32)	2.92	3.99 (2.65– 3.99)	<0.10 (<0.10–7.4)	17.76 (1.15–374)	–	7.5 (2.58–7.51)	>11.4
CRP (mg/dL) (range)	211.2 (146.3–367.3)	229.7 (16.5–501.8)	84.2 (84.2–364.5)	49.1 (29.8–148.1)	313.1 (131.7–313.1)	41.6	10.8 (10.8–28.6)	304 (67–387.8)	200.4 (200.4–309.9)	10 (7.7–374.3)	15.1 (15.1–384)	19.5 (14.0–19.5)	<5.0 (<5.0–226.8)	114.9
Ferritin (ng/mL) (range)	6,037	2,241 (1,884–5,176)	671 (503–790)	681 (257–791)	3,038	771	27 (27–34)	1,396 (341–1,396)	372 (349–372)	624 (423–1,749)	361 (36–4,942)	2,810	1,732 (931–1,732)	1,597
LDH (U/L) (range)	124 (124–228)	532 (478–678)	678 (272–678)	891 (320–1351)	928	413	193 (193–198)	511 (435–523)	407 (307–407)	907 (418–907)	356 (356–1,063)	371 (371 −446)	548 (246–548)	527.6
Fibrinogen (mg/dL) (range)	436	311 (253–311)	702 (676–702)	90 (65–436)	783	715	489 (489–599)	813	813	–	507 (507–785)	239	–	536.18
CK (U/L) (range)	12	637 (87–1,246)	19,247 (72–19,247)	52 (15–129)	765 (146–765)	168	42.0 (21.0–53.0)	177 (74–511)	48	1,018 (400–3,475)	1,925 (1891–3,226)	163 (76–163)	9	1,866.3

*COVID-19, coronavirus disease 2019; Temp., Temperature; MAP, mean arterial pressure; HR, heart rate; RR, respiratory rate; SpO2, pulse oximetry; WBC, white blood cells; AST, aspartate transaminase; ALT, alanine transaminase; CRP, C-reactive protein; LDH, lactate dehydrogenase; CK, creatinine kinase*.

### Outcome Measures

Ischemic strokes were predominantly cortical (84.6%), in the distribution of the middle cerebral artery (76.9%), followed by the posterior cerebral artery (23%). Stroke etiology was classified as cryptogenic and/or ESUS in 53.8% (*n* = 7), cardioembolic in 23% (*n* = 3), large artery atherosclerosis in 15.3% (n = 2), and small vessel disease in 7% (*n* = 1). ESUS was suspected in 71.4% of “Neuro” compared to 33.3% of “COVID.” Overall, 60% (*n* = 3) of patients with evidence of large vessel occlusion (LVO) underwent endovascular thrombectomy. While the “COVID” group had more LVO (50%), more patient in the “Neuro” group (42.8 vs. 16.6%) received acute stroke interventions, with delays in identification of AIS symptoms in the “COVID” group attributed to masking of symptoms by the COVID-19 systemic manifestations. Therapeutic anticoagulation was initiated in 38.4% (*n* = 5) patients due to concerns of hypercoagulable state and in 7% (*n* = 1) due to atrial fibrillation. The median NIHSS at discharge for the COVID AIS cohort was 11 (IQR 4–23), with median mRS of 4 (IQR 3–4). Favorable outcome with discharges to home or to acute rehabilitation facilities was seen in 61.5% (*n* = 3) Two patients (15.3%) expired from COVID-19 complications, and two (15.3%) required long term facility care. One patient remains hospitalized (Table 1).

### Univariate and Logistic Regression Analysis

Except for DM type 2 which was more prevalent in the COVID-19 AIS group (64.2 vs. 24.5%, *p* 0.008), patients were overall equally balanced for age, sex and comorbidities with the non-COVID stroke cohorts from 2019 and 2020. The COVID-19 AIS group had more Latinos compared to both historical 2019 and 2020 cohorts (46.1 vs. 9%, *p* 0.0036 and 46.1 vs. 9.5%, *p* 0.0075). The percentage of African-American patients in all groups was similar (31 vs. 32%, *p* 0.989 and 31 vs. 36%, *p* 0.766). While the median admission NIHSS was not different among the COVID-19 AIS and the non-COVID-19 stroke patients in 2020 and 2019, the discharge NIHSS was significantly higher [11 (IQR 4–23) vs. 3 (IQR 2–13), *p* 0.036 and 11 (IQR 4–23) vs. 4 (IQR 1–11), *p* 0.042]. There was a trend toward less alteplase administration in the COVID-19 AIS patients though no statistically significant (7.1 vs. 20.7%, *p* 0.435 and 7.1 vs. 27.2%, *p* 0.178). COVID-19 AIS cohort had worse mRS > 2 at discharge (78.5 vs. 47.16%, *p* 0.047 and 78.5 vs. 40.9%, *p* 0.010) (Table 3), even after correction for age and sex in a logistic regression model [*p* 0.046, OR 3.82, (CI 1.02–14.3)].

**Table 3:** Comparison of acute ischemic stroke patients with COVID-19 infection with acute ischemic stroke patients without COVID-19 infection admitted in the same time frame of March 1, 2020–April 30, 2020, and March 1, 2019–April 30, 2019.

	**1. COVID-AIS group (*N* = 13)**	**2. AIS group 2020 (*N* = 53)**	**3. AIS group 2019 (*N* = 88)**	***p*-value 1 vs. 2**	***p*-value 1 vs. 3**
Age (mean)	61.6	63	68	0.935	0.096
Male Sex (%)	46.1	52.8	51.1	0.569	0.597
Race/Ethnicity (%)					
Latino	46	9.5	9	**0.0075**	**0.0036**
African American	31	32	36	0.989	0.766
Comorbidities (%)					
CAD	15.3	15	19.3	0.664	0.738
DM	69.2	24.5	38.6	**0.006**	0.069
HTN	69.2	52.8	75	0.549	0.522
HLD	30.7	39.6	42	0.548	0.397
CHF	7.6	3.7	13.6	0.992	0.689
Prior ischemic stroke	0	13.2	17	–	–
PAD	15.2	0	7.9	–	0.603
Alcohol/drug abuse	0	11.3	6.8	–	–
Tobacco abuse	0	22.6	22.7	–	–
Obesity (BMI > 30)	15.2	28.3	32.9	0.496	0.219
Admission NIHSS (median) (IQR)	16 (4–23)	8 (3–19)	7 (2–16)	0.081	0.089
Discharge NIHSS (median) (IQR)	11 (4–23)	3 (2–13)	4 (1–11)	**0.036**	**0.042**
Stroke Etiology (%)					
Cryptogenic and/or ESUS	53.8	47.1	30.6	0.763	0.121
Cryptogenic and/or ESUS + cardio-embolism	76.9	67.9	61.3	0.519	0.244
IV-tPA (%)	15.3	20.7	27.2	0.435	0.178
EVT (%)	23	20.75	26.1	0.999	0.507
Discharge mRS (median) (IQR)	4 (3–4)	3 (1–4)	3 (1–4)	0.050	0.063
Discharge mRS >2 (%)	76.9%	47.16	40.9	**0.047**	**0.010**

*COVID-19, coronavirus disease 2019; AIS, Acute ischemic stroke; CAD, coronary artery disease; DM, Diabetes mellitus type 2; HTN, Hypertension; HLD, Hyperlipidemia; CHF, congestive heart failure; PAD, peripheral artery disease; BMI, body mass index; NIHSS, National Institutes of Health Stroke Scale; ESUS, Embolic stroke of unknown source; IV-tPA, intravenous-tissue plasminogen activator; EVT, Endovascular thrombectomy; mRS, modified Rankin Scale. The bold numerical values indicates statistically significant*.

## Discussion

In this single-center retrospective observational study, we identified 13 patients with AIS and concomitant COVID-19. Approximately 2.0% of all COVID-19 patients at our institution were diagnosed with AIS, a percentage higher than previously reported in the literature from the USA (12) but similar to that from Wuhan, China (5). The mean age of patients with AIS and COVID-19 was 61.6, without significant sex predilection. Unlike the data emerging out of New York (12), there was a higher percentage of Latinos and African Americans in our cohort (76.8%), highlighting the racial disparity of COVID-19 in our Metropolitan city. Several studies have highlighted the disproportionate burden of this disease on these communities. Social and economic disparities, less access to healthcare along with genetic factors associated with more potent thrombo-inflammatory response may have contributed to higher infection rate and worse outcome in Latinos and African-Americans (13, 14). The majority of our patients had the severe or critical form of COVID-19, which re-iterates the prior published findings of high prevalence of neurological complications seen in this group (2). Also, the trend toward cortical strokes with etiological classification as ESUS reflects the coagulopathy and potential causal link between COVID-19 and stroke (12).

The delay in conventional stroke interventions especially amongst patients who developed AIS while receiving treatment for COVID-19 may be explained by the masking of acute stroke symptoms by the viral illness, delay in stroke symptoms recognition, and/or use of anticoagulation at the time of evaluation.

Several potential mechanisms can lead to a stroke in the setting of COVID-19. Angiotensin-converting enzyme which is the target site of SARS-CoV2 is expressed by cells of the nervous system. This renders the brain at risk of direct endothelial cell infection and diffuse endothelial inflammation (15). COVID-associated coagulopathy which is likely the result of intense inflammatory response, can lead to increased thrombotic complications including ischemic stroke (16). Cardiac involvement is also a prominent feature of COVID-19, leading to stress cardiomyopathy, direct myocardial injury, and arrhythmias with potential increased risk of ischemic stroke (17). Lastly, prolonged hospitalization and dysautonomia may lead to ischemic stroke especially in the setting of septic shock and hypotension (18).

## Strengths and Limitations

Our study provides a detailed description of patients with COVID-19 and AIS and highlights the racial disparity and poor outcomes associated with this highly contagious viral infection. This study also highlights that despite COVID-19 affecting elderly patients more severely, increased risk of AIS in COVID-19 is independent of age. Comparison with current and historical cohorts suggests a direct causal link of COVID-19 and AIS highlighting the importance of checking for COVID-19 in patients with ESUS and/or cryptogenic stroke mechanisms.

Our study has several limitations with its small size, retrospective approach, and lack of long term follow up and outcome. We also suspect that the incidence of AIS is much higher as many patients with the infection may have succumbed to the disease before identification of the stroke symptoms, or may not have been evaluated by the neurology service, and thus the neurological symptoms may not have been captured.

## Conclusion

In summary, ischemic stroke in COVID-19- tend to be more severe, mainly cortical, may occur independent of common vascular risk factors, does not have sex predilection and can affect younger population also. AIS in COVID-19 was more commonly seen in Latino and African American communities by our group, a reflection of the health care disparity and limited access to care among the minority population.

## Data Availability Statement

The raw data supporting the conclusions of this article will be made available by the authors, without undue reservation.

## Ethics Statement

The studies involving human participants were reviewed and approved by Rush University Institutional review board. Written informed consent for participation was not required for this study in accordance with the national legislation and the institutional requirements.

## Author Contributions

PG and PP contributed equally as first authors in the acquisition of data, interpretation of data, and manuscript writing. ID and JC contributed equally as last authors in the interpretation of data and critical revision of the manuscript for intellectual content. JH, RD, TT, DP, RG, NO, AV, and SJ contributed in design and acquisition of data. All authors take full responsibility for the credibility of the data and results of the manuscript.

## Conflict of Interest

The authors declare that the research was conducted in the absence of any commercial or financial relationships that could be construed as a potential conflict of interest.

## Abbreviations

SARS-CoV-2: severe acute respiratory distress syndrome virus
COVID-19: coronavirus disease 19
NIHSS: National Institutes of Health Stroke Scale.
